# Synergistic Improvement in Thermal Conductivity of Polyimide Nanocomposite Films Using Boron Nitride Coated Copper Nanoparticles and Nanowires

**DOI:** 10.3390/polym10121412

**Published:** 2018-12-19

**Authors:** Yongcun Zhou, Shihu Yu, Huan Niu, Feng Liu

**Affiliations:** 1 School of Materials Science and Engineering, Northwestern Polytechnical University, Xi’an 710072, Shaanxi, China; 2 State Key Laboratory of Electrical Insulation and Power Equipment, Xi’an Jiaotong University, Xi’an 710049, Shaanxi, China; yush0913@163.com (S.Y.); huanniu1@stu.xjtu.edu.cn (H.N.); 3 Electric Power Research Institute of Guangdong Power Grid Co. Ltd., Guangzhou 510080, Guangdong, China

**Keywords:** electrical properties, polymer matrix composite, thermal properties, coating

## Abstract

Electronic devices are increasingly dense, underscoring the need for effective thermal management. A polyimide (PI) matrix nanocomposite film combining boron nitride (BN)-coated copper nanoparticles (CuNPs@BN) and nanowires (CuNWs@BN) was fabricated by a flexible and fast technique for enhanced thermal conductivity and the dielectric properties of nanocomposite films. The thermal conductivity of (CuNPs-CuNWs)@BN/PI composite comprising 10 wt % filler loading rose to 4.32 W/mK, indicating a nearly 24.1-fold increase relative to the value obtained for pure PI matrix. The relative permittivity and dielectric loss approximated 4.92 and 0.026 at 1 MHz, respectively. The results indicated that the surface modification of CuNPs and CuNWs by introducing a ceramic insulating layer BN effectively promoted the formation of thermal conductive networks of nanofillers in the PI matrix. This study enabled the identification of appropriate modifier fillers for polymer matrix nanocomposites to improve electronic applications.

## 1. Introduction

Surface modifications of fillers in polymer matrix composite films attract increasing attention because of as-created core-shell structures possessing extraordinary features, including their use as biocatalysts, phase change materials, and other potential applications [1,2,3]. To comprehensively ameliorate compatibility and stability between polymer and filler interfaces while decreasing resistance of the thermal interface in polymer matrix composites, surface modifications seem to represent an optimal choice. However, surface modifications are mostly challenging, and the modification effect may not be as strong as expected, making it difficult to take advantage of most fillers’ superiority. Therefore, additional intrinsic parameters, including filler type, size, shape, and orientation, should be taken into consideration [4,5,6,7,8,9,10,11]. Of these, orientation represents an essential and easily amenable parameter, notably for one-dimensional tubes or wires and two-dimensional platelet fillers. Filler orientation in the heat flow direction is broadly used to yield an elevated thermal conductivity in polymer composites. Recent studies have shown that metal nanowires have a higher efficiency in improving thermal conductivity to decrease the percolation threshold in comparison with other nanostructures because of their inherent continuity and elevated aspect ratio [4,5]. Copper (Cu) is one of the most important metals in modern technology because of its inherent continuity, elevated thermal conductivity, and affordability. Therefore, copper nanoparticles (CuNPs) and copper nanowires (CuNWs) are excellent candidates for orientation fillers in polymer matrix composite films with high thermal conductivity [6,7,8,9,10,11,12,13]. Unfortunately, elevated electrical conductivity and relative permittivity hamper the application of copper-based materials for electronic packaging, which requires electrical insulation. Hence, the properties of copper, including elevated thermal conductivity and low relative permittivity, still need improvement for more applications as a filler of polymer matrix composites. Thermal resistance at the filler–filler or polymer–filler interface also plays a key role in polymer composites. Based on the above, we hypothesized that the thermal conductivity of composite films containing BN-coated copper orientation fillers could be increased via the reduction of interfacial thermal resistance, which still needs to be investigated.

In this study, a novel approach was developed through the surface modification of CuNPs and CuNWs using synthetic BN to generate flexible polyimide (PI) nanocomposites displaying elevated thermal conductivity but minimal relative permittivity. The BN nanolayer on CuNPs and CuNWs (denoted as CuNPs@BN and CuNWs@BN, respectively) formed a barrier that prevented the generation of conductive paths, effectively reducing the dielectric constant of composites. This “point-line” structure is considered to play a bridging role for copper nanoparticles with copper nanowires in enhancing the filler interaction to reduce interfacial thermal resistance. Furthermore, the multilayer-structure comprised CuNPs@BN and CuNWs@BN, providing a network efficiently improving the thermal conductivity of composites. The thermal management of PI nanocomposites according to the multilayer-structure comprising BN-coated CuNPs and CuNWs is scarcely studied.

## 2. Experimental Section

### 2.1. Materials

Copper nitrate trihydrate [Cu(NO_3_)_2_·3H_2_O], sodium hydroxide (NaOH), hydrazine hydrate (N_2_H_4_·H_2_O 85%), 2,4,6-Tris(dimethylaminomethyl)phenol, ethylenediamine (EDA), and Tris(hydroxymethyl)aminomethane were obtained from Sinopharm Chemical Reagent Co., Ltd. (Shanghai, China). Dopamine (DA, 98%), polyvinylpyrrolidone (PVP, MW ≈ 40,000, powder), boric acid (H_3_BO_3_, 99%), urea [CO(NH_2_)_2_, 96.5%], tetraethylorthosilicate (TEOS, 99.0%), and ammonium hydroxide (NH_4_OH, 28 wt %) were manufactured by Alfa Aesar Co. (Beijing, China). Polyimide (PI) and the *N*,*N*-dimethylacetamide (DMAc) solvent were purchased from Sigma (Shanghai, China). Copper (Cu, ~200 nm) nanopowder was purchased from Beijing DK Nano Technology Co. Ltd. (Beijing, China). The remaining chemicals were of analytical grade.

### 2.2. Synthesis of CuNPs@BN and CuNWs@BN

The synthesis of CuNWs was performed as previously reported [14]. The CuNWs@BN core-shell structure was prepared by direct impregnation. First, H_3_BO_3_ particles and 200 proof ethanol were mixed in a 500 mL round-bottom flask followed by sonication for 1 h in ambient conditions. After the H_3_BO_3_ had gradually been dissolved, urea was added under ultrasonication for 1 h. The resulting taupe powder was vacuum-dried at 60 °C for ≥12 h. After purging with nitrogen gas for ≥20 min, the furnace was heated to the operating temperature at 5 °C/min, with ammonia flowing at 200 mL/min. Samples were heated at 900 °C for 3 h and cooled under ammonia. Finally, core-shell CuNPs@BN and CuNWs@BN nanopowders were obtained.

### 2.3. (CuNPs-CuNWs)@BN/PI Composite Film Preparation

**Figure 1:**
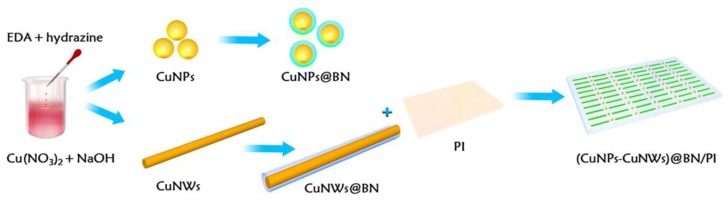
Preparation of multilayer-structured (CuNPs-CuNWs)@BN/PI film.

Figure 1 shows a schematic diagram outlining the experimental procedure for preparing (CuNPs-CuNWs)@BN/PI. Firstly, the PI matrix dissolution was carried out in acetone, followed by the CuNPs@BN and CuNWs@BN dispersion under ultrasonication to homogeneity. Then, the samples were mixed and vacuum-dried at 60 °C. Subsequently, the obtained mixtures were spread onto glass with thermal imidization under vacuum at 80 °C for 2 h followed by 150, 200, 250, and 350 °C for 1 h. Finally, (CuNPs-CuNWs)@BN/PI composites with a multilayer structure were obtained. As controls, PI composite films comprising untreated CuNPs and CuNWs were obtained as described above.

### 2.4. Characterization

CuNPs, CuNWs, CuNPs@BN, and CuNWs@BN were assessed for microstructure and morphology by high-resolution transmission electron microscopy (HRTEM; JEOL JEM-1230, Tokyo, Japan) and scanning electron microscopy (SEM; JEOL 7401F, Tokyo, Japan). SEM was performed with a voltage of 3 kV, with the specimens sputter-coated using a thin layer of gold. Thermal conductivity assessment was performed on a thermal constant analyzer (HOT DISK TPS-2500s, Uppsala, Sweden). Bulk densities of specimens were determined by the water displacement method on an electronic densimeter (METTLER TOLEDO, XPE205, Zurich, Switzerland). Resistance indexes, such as volume resistivity and surface resistivity, were measured on a high-resistance meter (16339, Hewlett Packard, Palo Alto, CA, USA) in ambient conditions. Low frequency dielectric features for composite films were determined from 1 kHz to 1 MHz on a 4980-A impedance analyzer (Agilent, Palo Alto, CA, USA) with a 16034B dielectric test fixture. Triplicate experiments were carried out.

## 3. Results and Discussion

**Figure 2:**
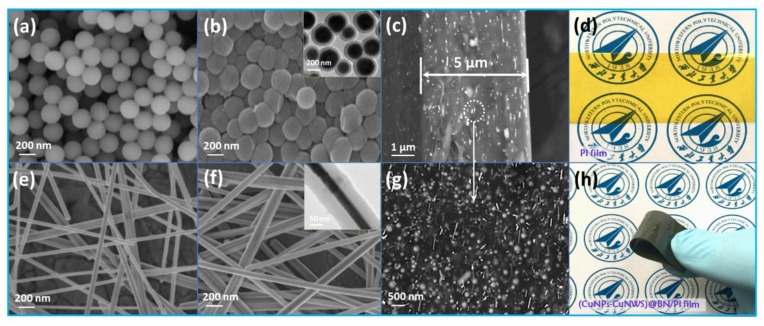
SEM micrographs of (**a**) CuNPs, (**b**) CuNPs@BN, (**c**) (CuNPs-CuNWs)@BN/PI film, (**e**) CuNWs, (**f**) CuNWs@BN, and (**g**) partial magnification of (**c**); TEM images of core-shell CuNPs@BN (inset of (**b**) and CuNWs@BN (inset of (**f**); optical micrographs of pure PI (**d**) and (CuNPs-CuNWs)@BN/PI (**h**) films.

SEM micrographs of CuNPs, CuNPs@BN, CuNWs, CuNWs@BN, and (CuNPs-CuNWs)@BN/PI are depicted in Figure 2a–c,e–g, respectively; TEM micrographs of core-shell CuNPs@BN and CuNWs@BN are displayed in insets of Figure 2b,f, respectively. Optical micrographs of pure PI and (CuNPs-CuNWs)@BN/PI films are found in Figure 2d,h, respectively. Optical micrographs of pure PI and (CuNPs-CuNWs)@BN/PI films are found in Figure 2d,h, respectively. The average diameter of untreated CuNPs approximated 200 nm, while the obtained CuNWs were 50 nm in diameter and 2–4 μm in length (Figure 2a,e). The BN shell was scrupulously coated on CuNPs and CuNWs upon surface modification, which showed overtly larger diameters post-treatment in comparison with CuNPs and CuNWs (Figure 2b,f, and insets of Figure 2b,f). The thickness of the BN nanoshell was about 50 nm, which promoted heat transfer in composite films. Figure 2c,g depict SEM micrographs of (CuNPs-CuNWs)@BN/PI composites with 10 wt % filler loading. The (CuNPs-CuNWs)@BN/PI composite films were approximately 5 μm thick; partial enlargement (Figure 2g) indicated a good CuNPs@BN and CuNWs@BN dispersion in the PI matrix, resulting in a “point-line” structure that promoted the generation of a thermal conduction network. Figure 2d shows that the pure PI film was yellowish and transparent, and the Northwestern Polytechnical University logo underneath was clearly visible. Figure 2g shows that the (CuNPs-CuNWs)@BN/PI film was greenish-black and flexible. The films with pure PI to 10 wt % (CuNPs-CuNWs)@BN filler content showed 0% transparency.

**Figure 3:**
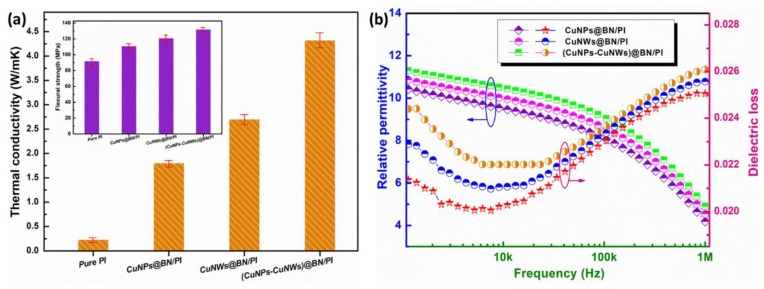
(**a**) Thermal conductivity values and flexural strengths (inset) of pure PI, CuNPs@BN/PI, CuNWs@BN/PI, and (CuNPs-CuNWs)@BN/PI nanocomposites assessed in ambient conditions; (**b**) relative permittivity and dielectric loss for CuNPs@BN/PI, CuNWs@BN/PI, and (CuNPs-CuNWs)@BN/PI films at frequencies between 1 kHz and 1 MHz.

Figure 3a shows the thermal conductivity values for pure PI, CuNPs@BN/PI, CuNWs@BN/PI and (CuNPs-CuNWs)@BN/PI composites assessed in ambient conditions with 10 wt % filler loading. The thermal conductivity values for the four films progressively increased with the filler type. The thermal conductivity of (CuNPs-CuNWs)@BN/PI was elevated compared with those of the remaining three films, peaking at 4.32 W/mK with 10 wt % filler loading. It should be noted that the thermal conductivity of the CuNWs@BN/PI composite with 10 wt % was 2.61 W/mK, while that of the CuNPs@BN/PI composite was only 1.75 W/mK. The results suggested that the “point-line” structure has some advantages over the normal configuration. CuNWs’ dimensions promoted the generation of relatively more efficient networks for thermal conductivity in comparison with CuNPs. It is widely accepted that interfacial thermal resistance results from the contact between phases of the same or various constituents [15,16,17,18,19,20]. Copper nanoparticles and nanowires augment the contact areas in both cases, reducing the interfacial thermal resistance of (CuNPs-CuNWs)@BN/PI composites. These findings suggest that the thermal conductivity of composite films can be increased by decreasing the interfacial thermal resistance via filler modification and designing the microstructure of composites. Therefore, the filler morphology and composite microstructure are critical in defining the thermal conductivity of polymer-based composite films. The Figure 3a inset shows the impacts of three fillers on the flexural strengths of composites and pure PI. The flexural strength was 128.0 MPa for (CuNPs-CuNWs)@BN/PI with 10 wt % filler loading, indicating a 39.1% increase relative to the value obtained for pristine PI matrix (92.0 MPa). These results indicated that the appropriate filler loading with an optimal surface modification efficiently promotes stress relaxation under external forces, thus improving flexural strength in composite films. Relative permittivity and dielectric loss for CuNPs@BN/PI, CuNWs@BN/PI, and (CuNPs-CuNWs)@BN/PI composites at 1 kHz and 1 MHz for BN and PI, respectively, are depicted in Figure 3b; the three films had comparable changes in both parameters with increasing frequency. The relative permittivity and dielectric loss obtained for (CuNPs-CuNWs)@BN/PI were 4.92 and 0.026, respectively, at 1 MHz with 10 wt % filler loading. (CuNPs-CuNWs)@BN/PI showed a relative permittivity of 4.92, which is lower and more suitable for substrates and packaging applications. The dielectric loss also remained low. Generally speaking, the relative permittivity of a polymer matrix composite increases with filler loading, with the premise that the fillers have the same morphology. However, at a high filler loading, relative permittivity might be reduced by voiding from inadequate filler packing and poor preparation methods. In the present work, the effects on dielectric features could result from space charge polarization in copper nanoparticles and the network of nanowires generated via junctions among particles, as well as interactions at the metal copper conductor to BN ceramic insulator interface. Dielectric loss is mostly composed of polarization and conduction losses. Conduction loss results from the charge flowing through the composite and is determined by the composite’s electric conductivity [21,22]. The above findings suggest the current technique used in BN coating for nanocomposite production has high efficacy in disrupting aggregated nanoparticles and generating efficient heat conduction networks, while yielding optimal relative permittivity for composite films. Thermal conductive and electrically insulating polymer composite films described in the literature are summarized in Table 1, which demonstrates the superiority of the present work in increasing thermal and dielectric features. Polymer composite films with the “point-line” structure showed an elevated thermal conduction and electrical insulation at low filler loading compared with previously published composite films. The above results indicated that modifying the surfaces of CuNPs and CuNWs via the introduction of a ceramic insulating BN nanolayer efficiently promotes the generation of thermal conductive nanofiller networks in the PI matrix. The 50 nm-thick BN nanolayer decreased the discrepancy between the high and low moduli of CuNP and CuNW fillers and PI matrix, respectively, reducing thermal interfacial resistance.

## 4. Conclusions

Overall, a (CuNPs-CuNWs)@BN/PI composite film combining core-shell CuNPs@BN with CuNWs@BN was generated using a flexible and fast method for increased thermal conductivity and dielectric features. The (CuNPs-CuNWs)@BN/PI film at 10 wt % filler loading showed a thermal conductivity reaching 4.32 W/mK, indicating a nearly 24.1-fold increase relative to pure PI matrix. The relative permittivity and dielectric loss approximated 4.92 and 0.026 at 1 MHz, respectively. The maximum flexural strength of the (CuNPs-CuNWs)@BN/PI composite increased to 128.0 MPa. These results indicated that the morphology of fillers and the microstructure of composites are critical for the thermal conductivity in polymer-based composite films. The present study provided novel insights into designing thermally conductive polymer-based composite films, which could be applied in next-generation electronic packaging.

## Figures and Tables

**Table 1:** Thermal conductivity and dielectric features of the composites generated in this and other studies.

Filler	Content	Thermal Conductivity (W/mK)	Electrical Conductivity (S/cm)	Dielectric Constant	Reference
LPMs	25 vol %	0.25	2 × 10^−12^	–	[6]
CuNWs	0.9 vol %	2.46	0.04	–	[7]
Al_2_O_3_@PDA	30 vol %	0.59	–	4.06	[8]
CuNWs@SiO_2_	15 wt %	1.1	1.13 × 10^−6^	–	[9]
CuNWs@TiO_2_	2 vol %	1.16	8.1 × 10^−8^	–	[10]
CuNWs@PDA	3.1 vol %	2.87	–	–	[11]
(CuNPs-CuNWs)@BN	10 wt %	4.32	7.5 × 10^−7^	4.92	This work
